# Gliadin Induces Neutrophil Migration via Engagement of the Formyl Peptide Receptor, FPR1

**DOI:** 10.1371/journal.pone.0138338

**Published:** 2015-09-17

**Authors:** Karen M. Lammers, Marcello Chieppa, Lunhua Liu, Song Liu, Tatsushi Omatsu, Mirkka Janka-Junttila, Vincenzo Casolaro, Hans-Christian Reinecker, Carole A. Parent, Alessio Fasano

**Affiliations:** 1 Department of Pediatrics, Mucosal Immunology and Biology Research Center, Massachusetts General Hospital East, Charlestown, Massachusetts, United States of America; 2 Lymphocyte Biology Section, Laboratory of Immunology, National Institute of Allergy and Infectious Diseases (NIAID), National Institutes of Health (NIH), Bethesda, Maryland, United States of America; 3 Laboratory of Cellular and Molecular Biology, Center for Cancer Research, NCI, NIH, Bethesda, Maryland, United States of America; 4 Department of Gastroenterology, Massachusetts General Hospital, Boston, Massachusetts, United States of America; Ludwig-Maximilians-Universität, GERMANY

## Abstract

**Background:**

Gliadin, the immunogenic component within gluten and trigger of celiac disease, is known to induce the production of Interleukin-8, a potent neutrophil-activating and chemoattractant chemokine. We sought to study the involvement of neutrophils in the early immunological changes following gliadin exposure.

**Methods:**

Utilizing immunofluorescence microscopy and flow cytometry, the redistribution of major tight junction protein, Zonula occludens (ZO)-1, and neutrophil recruitment were assessed in duodenal tissues of gliadin-gavaged C57BL/6 wild-type and Lys-GFP reporter mice, respectively. Intravital microscopy with Lys-GFP mice allowed monitoring of neutrophil recruitment in response to luminal gliadin exposure in real time. *In vitro* chemotaxis assays were used to study murine and human neutrophil chemotaxis to gliadin, synthetic alpha-gliadin peptides and the neutrophil chemoattractant, fMet-Leu-Phe, in the presence or absence of a specific inhibitor of the fMet-Leu-Phe receptor-1 (FPR1), cyclosporine H. An irrelevant protein, zein, served as a control.

**Results:**

Redistribution of ZO-1 and an influx of CD11b^+^Lys6G^+^ cells in the *lamina propria* of the small intestine were observed upon oral gavage of gliadin. *In vivo* intravital microscopy revealed a slowing down of GFP^+^ cells within the vessels and influx in the mucosal tissue within 2 hours after challenge. *In vitro* chemotaxis assays showed that gliadin strongly induced neutrophil migration, similar to fMet-Leu-Phe. We identified thirteen synthetic gliadin peptide motifs that induced cell migration. Blocking of FPR1 completely abrogated the fMet-Leu-Phe-, gliadin- and synthetic peptide-induced migration.

**Conclusions:**

Gliadin possesses neutrophil chemoattractant properties similar to the classical neutrophil chemoattractant, fMet-Leu-Phe, and likewise uses FPR1 in the process.

## Introduction

Gluten-containing cereals, e.g. wheat, rye and barley, are an important part of the human diet. However, there is growing evidence that gliadin, the main component of gluten, is implicated in various disorders. Gliadin is identified as the trigger of the autoimmune enteropathy, celiac disease (CD) [1] and has been associated with wheat allergy [2]. In addition, several studies propose a connection between gluten intake and the development of other diseases in which immune-mediated phenomena are thought to play a preeminent role, such as irritable bowel syndrome prevalent diarrhea [3], non-celiac gluten sensitivity [4–6], type 1 diabetes [7], schizophrenia [8] and autism [9].

Evolutionally, gluten became part of the diet only recently, during the so-called “Neolithic revolution” period around 10,000 BC, when a nomadic life-style transitioned into one of settled farming and wheat was cultivated as a crop. Gliadin is a complex protein, rich in prolines and glutamines and, for this reason, cannot be completely digested by the intestinal enzymes of the mammalian gastrointestinal tract [10]. Undigested gliadin peptides can be recognized by the intestinal immune system [1, 11–13].

An impaired intestinal barrier function may be part of the pathogenesis of gluten-associated gastrointestinal and systemic autoimmune disorders, such as CD and type 1 diabetes, perhaps reflecting genetic predisposition [7, 14]. Our previous studies have shown that gliadin can cause a transient increase of the intestinal permeability irrespective of disease status, though the length and duration of this effect are different in healthy individuals versus CD patients [15]. Similarly, when tested on C57BL/6 mice duodenal tissues, gliadin caused increased gut mucosa permeability [16]. In the latter study, we identified two alpha-gliadin motifs that were capable of modulating the intestinal barrier function by binding to the chemokine receptor, CXCR3, and subsequent zonulin-mediated disassembly of the inter-epithelial tight junction complex (TJ) [16] that in its intact shape regulates paracellular solute traffic [17].

The adaptive immune response that is triggered by gliadin has been extensively studied, but the paramount, as yet unresolved, question is how gliadin initiates the mucosal innate defense mechanism. We hypothesize that the gliadin-induced TJ disassembly and consequent loss of intestinal barrier function is one of the early and essential mucosal steps that eventually lead to the intestinal inflammation characteristic for CD and other gluten-associated disorders. Once the epithelial barrier is compromised, gliadin gains access to the *lamina propria* where it is recognized by the mucosal immune system. We and others have found that gliadin is capable of inducing the production of high titers of the neutrophil-activating and chemoattractant chemokine, interleukin (IL)-8, by immune cells [11, 12, 18]. The aim of this study was to understand the early host defense with a primary focus on the involvement of neutrophils following gliadin challenge.

## Materials and Methods

### Animals and human subjects

All mice were housed and handled according to the NIH and MGH Institutional Animal Care and Use Committee guidelines. The animal protocols, LI-31E and 2013N000013, were approved by the NIH and MGH Institutional Animal Care and Use Committee, respectively. For human blood samples, blood was collected from anonymous healthy donors enrolled in the NIH Blood Bank research program.

### Reagents

#### PT-gliadin preparation

Pepsin/Trypsin (PT)-digested gliadin is a commonly used and well-accepted stimulus in the field of celiac research. Gliadin was digested with pepsin and trypsin to mimic the physiological food digestion within the gastrointestinal tract *in vivo*. The digested gliadin suspension contains different sizes of peptides. The largest peptide that has been reported to exert an immunogenic reaction is a 33-mer peptide [1]. To prepare the digest, gliadin (Sigma) was dissolved in 0.2 M HCl. The mixture was first incubated with pepsin (Sigma) under continuous stirring for 18 h at room temperature. The peptic digest pH was adjusted to 7.4 to inactivate pepsin enzymatic activity. The mixture was then further digested by addition of purified trypsin (Sigma) overnight at 37°C. At the end of incubation, the solution was boiled for 30 min to inactivate trypsin enzymatic activity, divided into aliquots, freeze-dried and lyophilized. All preparations were commercially tested for endotoxin. In order to rule out the possibility of residual enzymatic activity interfering with the experiments, a PT-water control was prepared in parallel. PT-zein was prepared as an irrelevant protein control.

#### Synthetic alpha-gliadin peptides and other reagents

A library of twenty-five 20-mer, 10-mer overlapping, peptides was designed based on the amino acid sequence of alpha-gliadin by solid phase synthesis (S1 Table). N-formyl-Methionine-Leucine-Phenylalanine (fMet-Leu-Phe) and cyclosporine H were purchased from Sigma (St. Louis, MO). Unlabeled and FITC-labeled gliadin peptide, TLPAMCNVYIPPYCTIVPFG, and FITC-labeled fMet-Leu-Phe were synthesized and conjugated in the Peptide/Protein Core Facility (Massachusetts General Hospital, Charlestown, MA. Director: A. Khatri).

### Intravital microscopy experiments

Eight separate experiments were performed with Lys-green fluorescent protein (GFP) transgenic mice (NIH, Bethesda, MD). These C57BL/6 LYS-eGFP knock-in mice [19] were a gift from T. Graf (Albert Einstein University). The surgical procedure used to expose the luminal side of the duodenum was similar as previously described by Chieppa et al. [20] Lys-GFP transgenic mice were anesthetized using nebulized isoflurane (2% induction, 1% maintenance) in 30% O_2_/68–69% air. The abdomen was shaved, and a vertical incision was performed at the duodenal level. The proximal 7–8 cm of the small intestine was externalized from the peritoneum while carefully preserving blood flow and lymphatic vessel continuity. A 5-cm long intestinal loop was performed carefully avoiding blood flow interruption. The loop was injected with approximately 200 μl of PT-gliadin solution (1 mg/mL) or control solution (PBS). The region imaged was in the middle of the loop, immobilized on the disposable stage by using both a solid support and Vetbond tissue adhesive. The exposed region of the bowel was bathed in phenol red-free RPMI 1640 at 37°C, and the whole animal was placed within an enclosed microscope stage fitted with both an environmental heater and a thermal blanket. For the imaging we used an Olympus OV-100 and a Hamamatsu ORCA ER II CCD camera. A series of images of the immobilized loop in the Z plane was acquired every 15 seconds for 3 hours. For the imaging the mucosal side, the lumen of the duodenum was surgically exposed using a battery-operated cautery (AARON Medical, Clearwater, FL) and washed with PBS at 37°C to remove fecal material and mucus from luminal surfaces. Real-time intravital imaging of the mucosal side was conducted though a 20× water immersion lens (N.A. 1.0) using a Leica SP5 on a DM6000 stage fitted with a 16W IR laser (Chameleon; Coherent). Computational analysis was conducted using Imaris (BitPlane).

### Gavage experiments

Three separate experiments were performed with C57BL/6 wild-type or Lys-GFP mice. Mice were gavaged with PT-gliadin, PT-zein, PBS or drinking water.

#### Immunofluorescence microscopy

Two hours after gavage, mice were sacrificed and the duodenum was removed and fixed in 1% paraformaldehyde solution. The tissue was stained for TJ protein, zonula occludens (ZO)-1 (rabbit polyclonal) and analyzed using confocal microscopy (Leica SP5).

#### Flow cytometry

Small intestines collected from gavaged Lys6-GFP mice were divided into 4 segments and inverted onto polyethylene tubes (Becton Dickinson, Franklin Lakes, NJ). Mucus and epithelium were removed by 1 mM dithiothreitol (DTT; Sigma-Aldrich, St. Louis, MO) and 30 mM EDTA (Boston Bioproducts, Boston, MA), respectively. The lamina propria was therefore exposed and digested with 333 μg/mL Liberase TL (Roche, Indianapolis, IN) in Dulbecco’s Modified Eagle’s Medium (DMEM; Gibco Life Technology, Gaithersburg, MD) containing 5% fetal bovine serum (Atlanta Biologicals, Flowery Branch, GA) for 90 min at 37°C in a 5% CO_2_ humidified incubator. All cells digested from tissue were collected and passed through 70 μm nylon cell strainers (Fisher Scientific, Pittsburgh, PA) and then stained for flow cytometry. Digested cells were incubated in 10% serum and Fc receptor-blocking antibody (clone 93; catalog number 101320; BioLegend, San Diego, CA) for 15 min at 4°C and then stained with the following fluorescent-conjugated antibodies for 15 min at 4°C: PE/cy7-conjugated CD11c (HL3; 561022; BD Bioscience, Franklin Lakes, NJ), BV510-conjugated I-A/I-E (M5/114.15.2; 107635; BioLegend), APC/cy7-conjugated CD11b (M1/70; 101226; BioLegend), PE-conjugated CD103 (M290; 557495; BD Bioscience) and APC-conjugated Ly6G (1A8; 560599; BD Bioscience). Cells were analyzed on a LSRII flow cytometer or FACSCalibur flow cytometer (BD Bioscience) followed by FlowJo software (Tree Star).

### Neutrophil isolation

#### Murine neutrophils

Murine neutrophils were flushed out with a needle from the femur and tibia bone marrows of C57BL/6 mice (Jackson Laboratories) and collected in buffer (HBSS without Ca^2+^/Mg^2+^, supplemented with 0.1% BSA and 5 mM HEPES). Cell suspensions were filtered over a strainer and isolated by density gradient centrifugation over 52%/69%/78% Ficoll layers [21]. The interphase containing the neutrophil fraction was collected, washed and resuspended in PBS. Viability (> 95%) was assessed by trypan blue dye exclusion. Purity (98–99%) was determined using Hemacolor staining to examine the morphology of the nuclei of the cells with conventional light microscopy.

#### Human neutrophils

Heparinized venous blood was centrifuged over a Ficoll-Paque PLUS gradient (d = 1.077 g/ml, GE Healthcare, Boston, MA). The sedimented granulocyte/erythrocyte fraction was collected, and erythrocytes were lysed with cold lysis buffer (155 mM NH_4_Cl, 10 mM KHCO_3_, 0.1 mM EDTA). Cells were resuspended in HBSS without Ca^2+^/Mg^2+^ (Thermo Scientific, Tewksbury, MA), and viability and purity were assessed as described above. Viability of neutrophils was >95%, purity was 98–99%.

### Chemokine production experiments

Three to six separate experiments were performed with duodenal segments from C57BL/6 mice mounted in the microsnapwell system [16] and Caco-2 monolayers, respectively. Tissues or cells were incubated with PT-gliadin (1 mg/mL) or medium alone for various time points. Supernatants were collected and stored until assay. Supernatants were assayed for IL-8 or Keratinocyte Chemoattractant (KC), the mouse equivalent of human IL-8. Caco-2 supernatants were collected at 1, 2, 4 and 24 hours and assayed for IL-8 using an enzyme-linked immunosorbent assay (ELISA) according to the manufacturer’s protocol (BD Bioscience, New Jersey). Detection limit of the assay was 15 pg/mL. Microsnapwell supernatants were collected at the beginning of the experiments and at 2 hours of gliadin exposure, and the concentration of KC was determined (Cytokine Core Laboratory, University of Maryland, Baltimore, MD).

### Chemotaxis assays

#### EZ-TAXIScan chemotaxis assay

Neutrophils were counted and applied in the EZ-TAXIScan chemotaxis assay [22]. PT-gliadin (1 mg/mL) or fMet-Leu-Phe (100 nM) were used as stimuli, and, PT-digested water (1 mg/mL) and PT-zein (1 mg/mL) as a negative control. The EZ-TAXIScan chamber (Effector Cell Institute, Tokyo) was assembled as described by the manufacturer. Cell migration was recorded every 15 seconds for 30 minutes at 37°C in a humidified environmental chamber. Coverslips and chips used in the chamber were coated with 1% BSA at room temperature for 1 hour. All glass coverslips were ultrasonicated and washed before use. Cell migration analysis was conducted with MATLAB software. Cell tracks were subsequently used to compute the chemotaxis index (CI) and speed for each tracked cell at each time step, as well as the average CI and average speed for each experiment. Each image shows a representative section of the final frame in the experiment, plus the locations of all cells identified and tracked in the section in all frames.

#### Transwell migration assay

This series of experiments was performed to test specificity of migration to PT-gliadin. Isolated human neutrophils were stained with Calcein AM according to the manufacturer’s protocol (Life Technologies, Carlsbad, CA). This fluorescent dye can serve as a marker for cell viability as it is transported through the membrane into live cells, and it allows reading neutrophil migration by a fluorometer. Calcein AM-stained neutrophils (5x10^5^/well) were added at the upper compartment of transwells (3 μm filter pore size), and medium alone or PT-gliadin in doses ranging from 10 μg/mL to 1 mg/mL were added to the lower compartment. Transwells were incubated for 45 minutes at 37°C. At the end of incubation, neutrophil migration to the lower compartments was calculated by reading the optical density-values of the samples and standard (serial dilution from 500,000 to 0 of calcein AM-stained neutrophils) by a fluorometer (excitation at 492 nm, emission at 520 nm). Data are expressed as percentage of migrated neutrophils.

### FPR1 binding and agonist assays

HEK293T cells were transfected with human FPR1 according to the manufacturer’s protocol (Origene cDNA ORF Clone, OriGene Technologies, Rockville, MD). Transfected and non-transfected cells were incubated with FITC-labeled gliadin peptide, TLPAMCNVYIPPYCTIVPFG, and/or increasing concentrations of fMet-Leu-Phe. Binding kinetics were evaluated by flow cytometric analysis. Specificity of binding of the synthetic gliadin peptide to FPR1 was assessed with the use of FPR1-transfected HEK293T cells. The transfected cells were incubated with increasing concentration of FITC-labeled gliadin peptide (100 nM to 250 μM) or FITC-labeled fMet-Leu-Phe (1 nM to 1 μM). Binding was determined by flow cytometry and expressed as mean fluorescence intensity (MFI). The specific binding was calculated as MFI_FPR1_—MFI_mock_. In the competitive binding assay experiments, transfected cells were exposed to a fixed concentration (25.6 μM) of the FITC-labeled gliadin peptide and increasing concentrations of fMet-Leu-Phe (10 nM to 2 mM) or a fixed concentration (100 nM) of FITC-labeled fMet-Leu-Phe and increasing concentrations of unlabeled gliadin peptide (1 nM to 500 μM).

### Statistical analysis

Differences between 2 groups were compared using the Student’s Test (for quantification of CD11b+Ly6G+ recruitment in the gavage experiments) or Mann-Whitney *U* test (for quantification of neutrophil recruitment in the intravital experiments and for chemotaxis assays). *P* values <0.05 were considered significant.

## Results

### *In vivo* intestinal luminal injection of PT-gliadin induces an immediate and substantial recruitment of neutrophils

**Fig 1:**
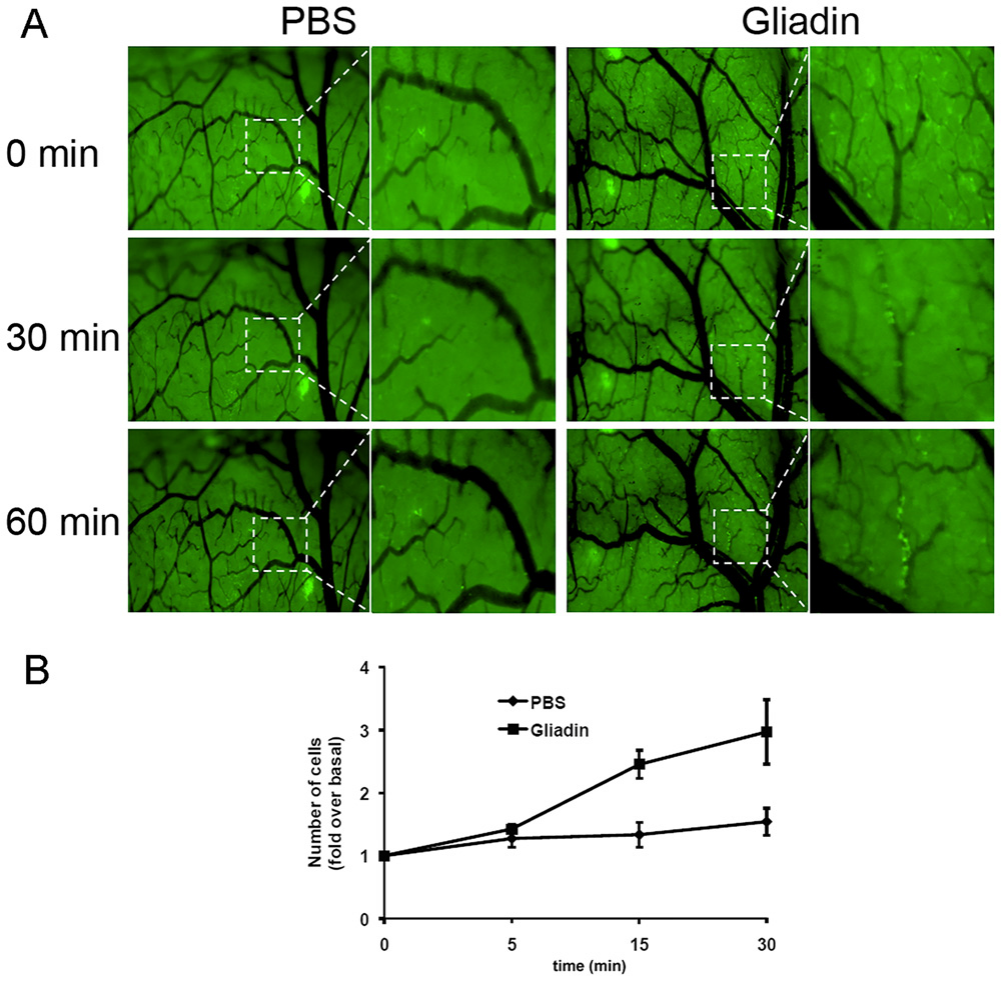
*In-vivo* intestinal luminal injection of PT-gliadin induces an immediate and considerable neutrophil migration. (A) Image stills at different time points from S1 movie. Intravital microscopy of the duodenum of Lys-GFP mice showed a rapid extravasation and recruitment of neutrophils (bright green) within 1 hour after PT-gliadin luminal administration, which was absent in mice treated with the same volume of PBS. Data are representative of results obtained from eight independent experiments (*n* = 8 for PT-gliadin, *n* = 8 for PBS). (B) Quantitative analysis of neutrophil recruitment in response to intestinal luminal PT-gliadin challenge. The green fluorescent channel in the original images was extracted, converted and processed into 16 bit binary images using the Image J software. The number of bright spots, which represents the number of neutrophils in the movies, was calculated with Image J using the Analyze Particles function. Neutrophil recruitment started immediately upon intestinal gliadin exposure, became significant after 15 minutes (*P* = .006) and increased further over time (*P* = .019 at *t* = 30 minutes).

Increased intestinal permeability favors the access of gliadin and other macromolecular antigens from the intestinal lumen into the *lamina propria* where the host, by interpreting them as danger signals, will respond with first-line defense mechanisms such as neutrophil recruitment to the site of exposure. To understand the dynamics of neutrophil recruitment upon pepsin/trypsin digested (PT)-gliadin exposure, we performed *in vivo* intravital microscopy using Lys-GFP transgenic mice [20]. In these mice GFP is expressed selectively in neutrophils and macrophages. We first imaged neutrophil recruitment from the luminal side of the intestine. Even if limited by the acquisition field, we observed that numerous green fluorescent cells were recruited following PT-gliadin administration (data not shown). We then visualized a larger field of the duodenum by imaging from the serosal side of the intestine. Shortly after PT-gliadin administration we observed that green fluorescent cells were slowing down, rolling along the endothelium and accumulating into the tissue (Fig 1, S1 movie-gliadin). Importantly, recruitment started within 30 minutes after injection of PT-gliadin, consistent with the timing of PT-gliadin-induced increase in intestinal permeability [16]. Since the surgical procedure could have been responsible for cell recruitment, parallel experiments were performed in mice treated with the same volume of PBS as negative control. No significant recruitment of green fluorescent cells was observed in these mice (Fig 1, S1 movie-control).

### PT-gliadin gavage induces the redistribution of zonula occludens-1 and an influx of CD11b+Ly6G+ cells in the lamina propria

**Fig 2:**
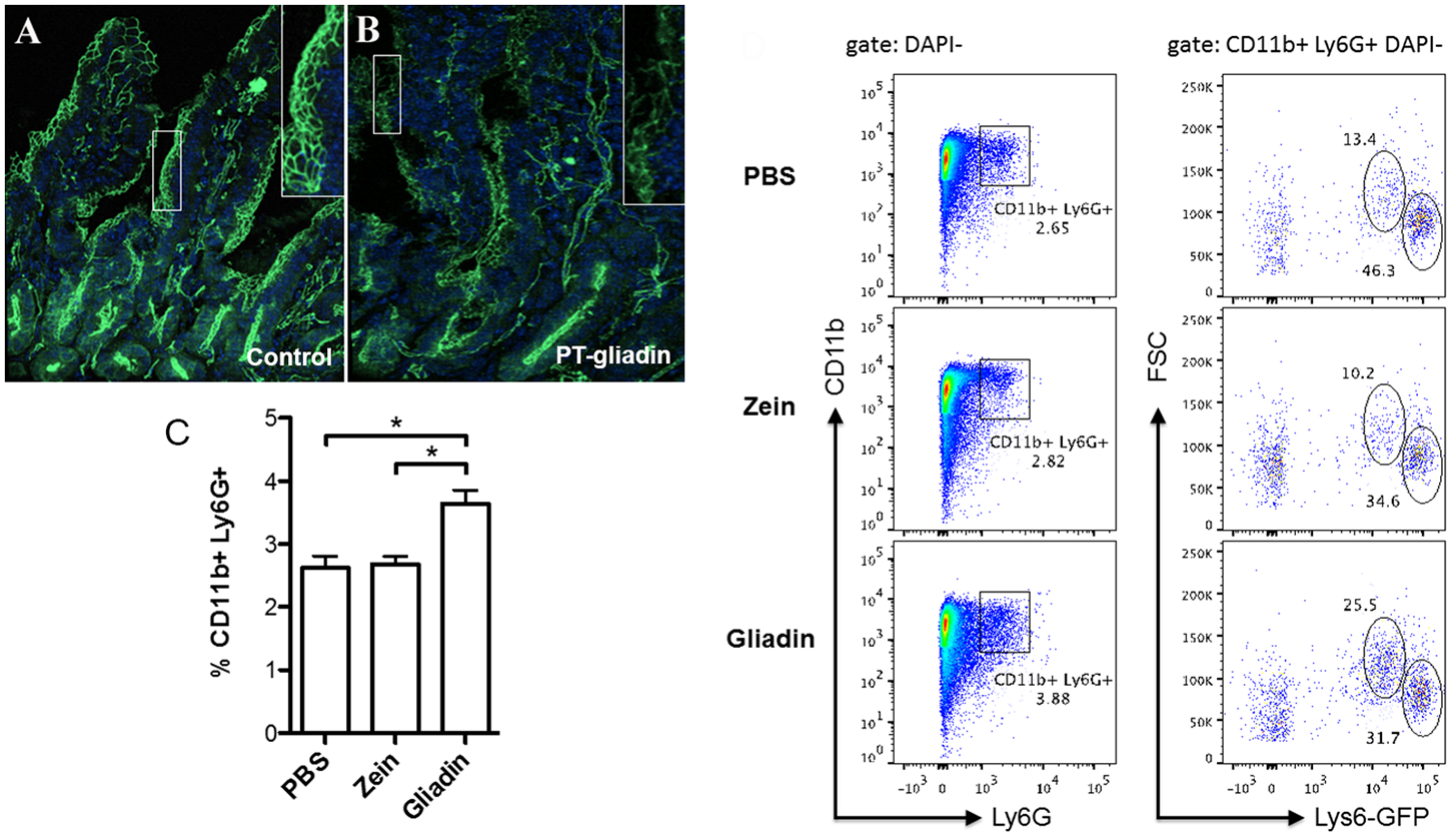
Gliadin causes redistribution of tight junction proteins, ZO-1, and recruits CD11b^+^Ly6G^+^ cells into the *lamina propria*. Representative images of C57BL/6 duodenum stained with antibodies specific for TJ protein, ZO-1 (green; A, B). Tissue sections were obtained from the duodenum of four mice 2 hours after gavage with PT-gliadin (1 mg/mL; B) or the same volume of drinking water (A) (*n* = 3). (C) Flow-cytometric analysis of CD11b^+^Ly6G^+^ cells isolated from the *lamina propria* of PBS-, PT-gliadin or PT-zein-gavaged Lys-GFP mice (cells were pooled from 2 mice per group). Compared to control and PT-zein, PT-gliadin ingestion induced an increased tissue influx of CD11b^+^Ly6G^+^ myeloid cells in the gut *lamina propria*. PBS *vs*. PT-gliadin, *P* = .0035; PT-zein *vs*. PT-gliadin, *P* = .0025. The graph represents data obtained from three separate experiments. (D) Representative dot plots of the flow cytometric analyses of the data depicted in graph 2C.

In this set of experiments we evaluated gliadin-induced TJ disassembly and neutrophil influx in the *lamina propria*. C57BL/6 wild-type mice were gavaged with PT-gliadin or drinking water. Two hours after gavage, duodenal tissues were prepared and stained for the major junctional protein, zonula occludens (ZO)-1. Compared to control mice, PT-gliadin-gavaged mice showed redistribution of ZO-1 (Fig 2A and 2B). In order to assess neutrophil recruitment in response to gliadin, Lys-GFP mice were gavaged with PT-gliadin, PT-zein or PBS control. Using flow cytometric analysis, we identified an increased influx of the CD11b^+^Ly6G^+^ cell subset, containing neutrophils and macrophages, in the *lamina propria* of PT-gliadin gavaged mice (Fig 2C and 2D). Mice gavaged with PBS or irrelevant PT-zein did not show recruitment of these cells, indicating that the influx of cells was a specific effect of gliadin exposure.

Together, these data show that the duodenal tissue of PT-gliadin-treated mice exhibits increased gut permeability and an elevated number of cells from the neutrophil/macrophage lineage.

### Addition of PT-gliadin to intestinal epithelial cells does not induce release or production of human IL-8/CXCL8 or murine KC/CXCL1

**Fig 3:**
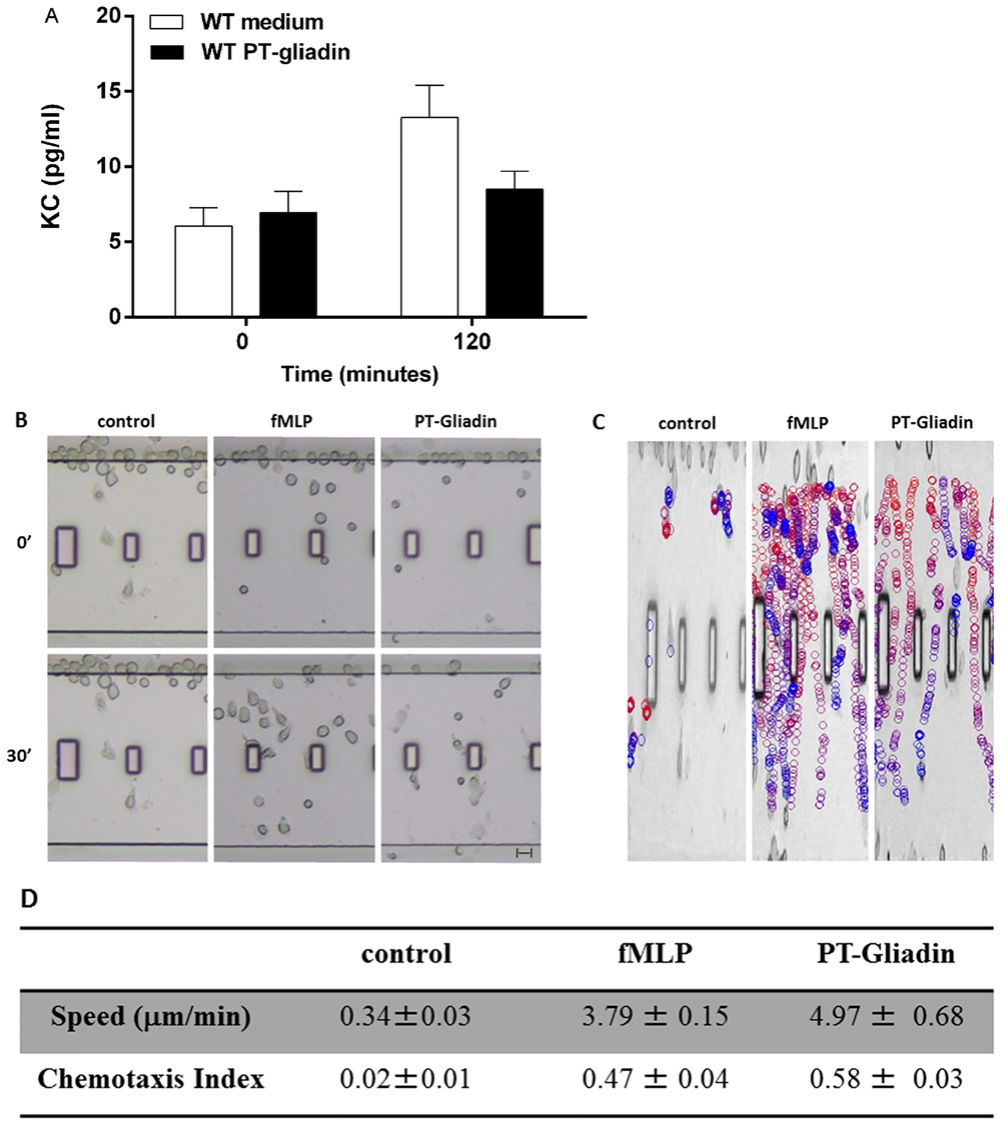
PT-gliadin attracts neutrophils but does not induce a rapid release of murine KC/CXCL1. (A) C57BL/6 intestinal segments did not show release of KC during the 2 hours incubation with PT-gliadin (*P* = NS). The graph represents data obtained from triplicate cultures from three mice. (B-D) EZ-TAXIScan chemotaxis of neutrophils isolated from the bone marrow of C57BL/6 mice toward medium alone, PT-gliadin or fMet-Leu-Phe (S2 movie). (B) Images from representative EZ-TAXIScan movies. (C) Image shows the paths of individual cells migrating to PT-water, PT-zein, PT-gliadin (1 mg/mL) or fMet-Leu-Phe (100 nM). The colors progress from red to blue as a function of time. (D) Quantitative analysis of all EZ-TAXIScan assay results of murine neutrophil chemotaxis in response to medium alone, PT-gliadin and fMet-Leu-Phe. The table depicts speed (μm/min) and the chemotactic index (considering speed and distance). Data are representative of results obtained from six independent movies.

Intestinal epithelial and immune cells can respond to a danger signal by producing chemokines that attract neutrophils to sites of damage [23]. The concept that chemokines acting on neutrophils can mediate gliadin-induced disease has been suggested in a number of studies showing neutrophil recruitment to the gut mucosa of CD patients within 24 hours after ingestion of gliadin [24, 25]. We and others [11, 12, 18] have previously shown that gliadin induces, in particular, a significant production of IL-8 (CXCL8), a potent neutrophil chemoattractant and activator. IL-8/CXCL8 is produced at high levels during immune activation by a range of immune and non-immune cells such as fibroblasts and epithelial cells [23]. To study whether the intestinal epithelium releases IL-8/CXCL8 within the first hours of PT-gliadin exposure, we added PT-gliadin to an *in vitro* human intestinal epithelial cell culture model (Caco-2 cells) as well as to an *ex vivo* microsnapwell model with mounted C57BL/6 intestinal segments and measured the production of IL-8/CXCL8 and its murine equivalent, keratinocyte chemoattractant (KC/CXCL1). No IL-8 release could be measured in either model within the 2 hour time frame of the experiment (Fig 3A and data not shown). These results suggest that the tissue influx of cells after PT-gliadin exposure *in vivo* is not mediated, at least in this initial instance, by IL-8 or KC production.

### PT-gliadin is a chemoattractant for murine and human neutrophils

The murine models showed that PT-gliadin, but not PT-zein, induced a rapid recruitment of CD11b^+^Ly6G^+^ cells into the gut mucosa. To explore the possibility that PT-gliadin itself could function as a chemoattractant for neutrophils, we made use of the EZ-TAXIScan chemotactic assay [22], which allows for the direct visualization of neutrophil chemotaxis to a given stimulus *in vitro*. We found that PT-gliadin induced chemotaxis of bone marrow-derived mouse neutrophils. Remarkably, PT-gliadin was at least as effective as the primary chemoattractant, fMet-Leu-Phe, at inducing chemotaxis (Fig 3B–3D, S2 movie).

**Fig 4:**
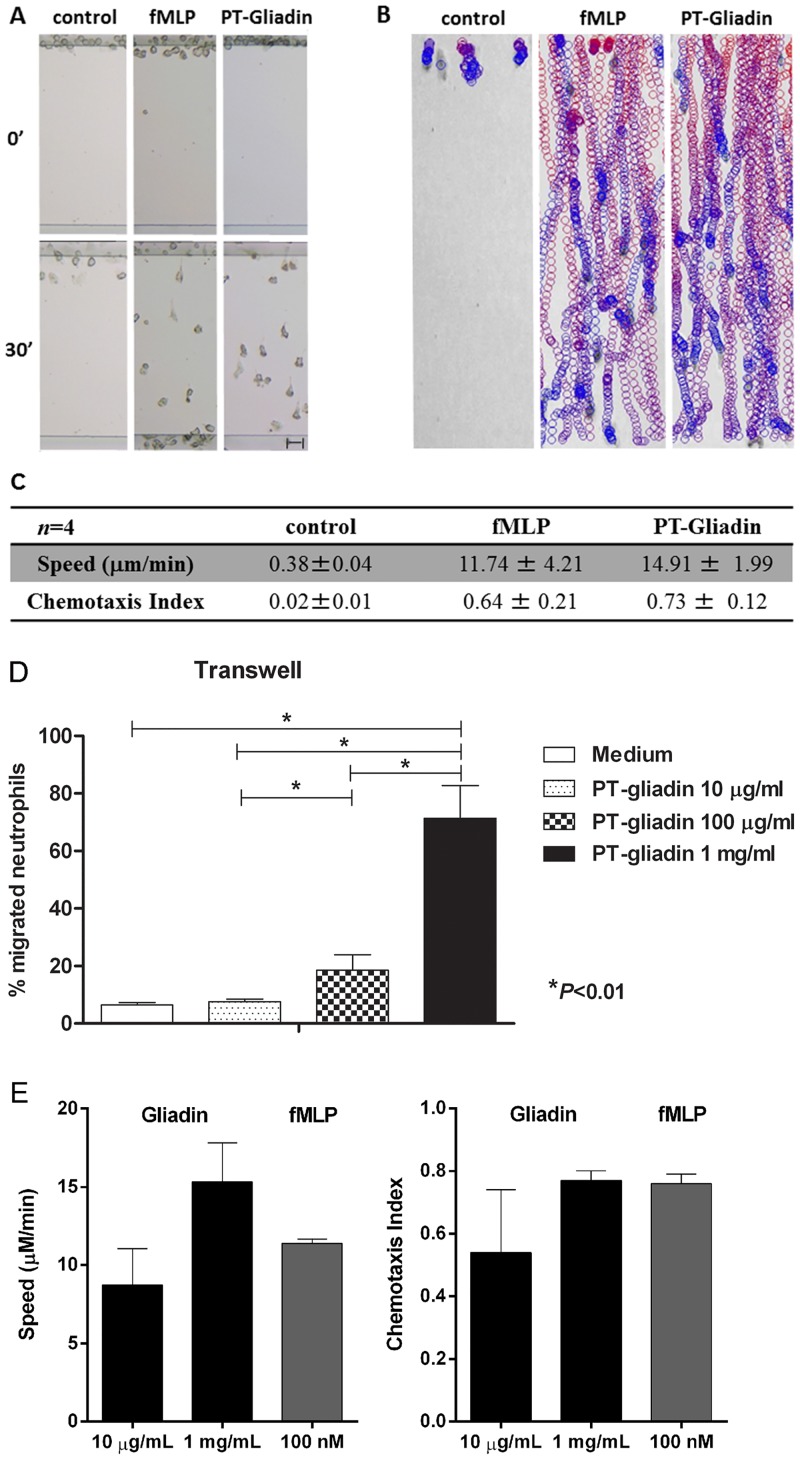
Human neutrophils migrate to fMet-Leu-Phe and PT-gliadin. (A-C) Data were obtained using the EZ-TAXIScan assay. (A) Images from representative EZ-TAXIScan movies (S3 movie). (B) Image shows the paths of individual cell migration to PT-water, PT-zein, PT-gliadin or fMet-Leu-Phe. The colors progress from red to blue as a function of time. (C) Quantitative analysis of all EZ-TAXIScan assay results of human neutrophil chemotaxis in response to PT-water, PT-zein, PT-gliadin and fMet-Leu-Phe. The table depicts speed (μm/min) and the chemotactic index (CI). PT-gliadin induced neutrophil chemotaxis with speed and CI similar to fMet-Leu-Phe (*P* = NS). Data are representative of results obtained from four independent movies. (D) PT-gliadin induced neutrophil migration in a dose-dependent manner in a Transwell chemotaxis assay (*P*<0.01). Data are obtained from six independent experiments. (E) PT-gliadin induced neutrophil migration in a dose-dependent manner in the EZ-TAXIScan assay. As a positive control, fMet-Leu-Phe was used. Data are obtained from three independent experiments.

To extend these observations to the human species, we conducted a similar set of experiments with neutrophils isolated from healthy volunteers in the same EZ-TAXIScan chemotaxis chamber. In accordance with the results obtained in mice, we found that PT-gliadin induced human neutrophil chemotaxis as effectively as fMet-Leu-Phe (Fig 4A–4C, S3 movie), whereas the controls PT-zein and PT-water failed to do so (S3 movie). Similar findings were obtained using a Transwell system (Fig 4D). PT-gliadin induced neutrophil migration in a dose-dependent manner with 1 mg/mL as the optimal dose (Fig 4D and 4E). These data show that PT-gliadin itself is a chemoattractant for neutrophils.

### fMet-Leu-Phe Receptor-1 (FPR1) mediates the chemotactic effects of PT-gliadin on neutrophils

The chemokine receptor, CXCR3, can act as a receptor for PT-gliadin [16]. The receptor is abundantly expressed on various immune cells but mostly on activated T cells [26]. Although the receptor is normally not expressed on circulating neutrophils, it was previously reported that neutrophils can acquire CXCR3 on their surface by release of the receptor from primary (azurophilic) granules in response to bacterial products [27]. We therefore determined whether neutrophil migration to PT-gliadin is mediated via an acquired surface expression of CXCR3. The chemotactic potential of PT-gliadin was assessed in neutrophils isolated from the bone marrow of C57BL/6 wild-type or CXCR3^-/-^ mice using the EZ-TAXIScan assay. We found that PT-gliadin caused a similar chemotactic response in neutrophils derived from either wild-type or mutated mice, showing that neutrophil recruitment is independent of PT-gliadin binding to CXCR3 (S4 movie).

Since gliadin and bacteria share a number of effects on the gut mucosa [16, 28], and given that of the three existing formyl peptide receptors (FPR-1, -2 and -3) only FPR1 recognizes fMet-Leu-Phe [29], the involvement of FPR1 in neutrophil chemotaxis to PT-gliadin was assessed using the EZ-TAXIScan assay after pretreating human neutrophils with cyclosporine H, a selective antagonist of FPR1 [30]. As expected, cyclosporine H treatment specifically abolished fMLP-mediated neutrophil chemotaxis while leukotriene B_4_ (LTB_4_)-mediated chemotaxis remained unaffected (Table 1). Remarkably, we found that PT-gliadin-induced chemotaxis was completely abrogated in the presence of cyclosporine H (Table 1). Together, these data suggest that PT-gliadin mediates neutrophil chemotaxis via engagement of FPR1.

**Table 1:** Blocking of FPR1 abrogates neutrophil migration to fMet-Leu-Phe and gliadin. Pretreatment of neutrophils with cyclosporine H, a specific inhibitor of FPR1, completely abrogated the neutrophil migration induced by PT-gliadin and fMet-Leu-Phe. As expected, LTB_4_ induced neutrophil chemotaxis was not affected by cyclosporine H.

**Peptide**	**Speed (μm/minute)**		***P*-value**	**Number of experiments**
	Control	Cyclosporine H		
fMet-Leu-Phe	11.39 ± 0.28	0.33 ± 0.02	*P*<.001	5
PT-gliadin	10.26 ± 0.38	0.31 ± 0.00	*P* = .001	5
LTB4	9.68 ± 0.30	9.23 ± 0.07	*P* = NS	5
**Peptide**	**Chemotaxis Index (CI)**		***P*-value**	**Number of experiments**
	Control	Cyclosporine H		
fMet-Leu-Phe	0.76 ± 0.03	0.03 ± 0.01	*P* = .001	5
PT-gliadin	0.51 ± 0.10	0.05 ± 0.01	*P* = .024	5
LTB4	0.51 ± 0.01	0.49 ± 0.01	*P* = NS	5

### Gliadin synthetic peptides induce chemokinetic responses

In order to exclude possible contamination of the PT-gliadin preparation with bacterial products and to find an alpha-gliadin motif responsible for the observed migration, an alpha gliadin synthetic peptide library consisting of twenty-five overlapping peptides [12] (Table 2) was tested for its capacity to induce neutrophil chemotaxis. Using the EZ-Taxiscan assay with human neutrophils, we found that thirteen out of the twenty-five peptides tested induced neutrophil migration with speeds ranging from 6.68 to 10.17 μm/min. However, compared with PT-gliadin, all thirteen peptides gave rise to a weaker directionally migration response, suggesting that the peptides generate a chemokinetic response (Table 2, S1 Fig).

**Table 2:** Synthetic peptides of the alpha-gliadin peptide library induce neutrophil migration. Alpha-gliadin synthetic peptides were tested for their capacity to induce neutrophil chemotaxis. Thirteen peptides induced neutrophil chemotaxis.

Peptide (20 ng/mL)	Chemotactic response	Speed (μm/minute)	Chemotaxis Index (CI)	Number of experiments
**MVRVPVPQLQPQNPSQQHPQ**	++	6.68 ± 1.92	0.30 ± 0.01	2
**PQNPSQQHPQEQVPLVQQQQ**	++	6.73	0.23	1
EQVPLVQQQQFLGQQQSFPP	-			1
FLGQQQSFPPQQPYPQPQPF	-			1
QQPYPQPQPFPSQQPYLQLQ	-			1
PSQQPYLQLQPFPQPQLPYL	-			1
**PFPQPQLPYLQPQPFRPQQP**	++	9.94 ± 0.09	0.21 ± 0.01	2
QPQPFRPQQPYPQPQPQYSQ	-			1
YPQPQPQYSQPQQPISQQQQ	-			1
**PQQPISQQQQQQQQQQQQQQ**	++	7.62	0.29	1
QQQQQQQQQQQQILQQILQQ	-			1
QLIPCMDVVLQQHNIAHGRS	-			1
**QQHNIAHGRSQVLQQSTYQL**	+++	10.17 ± 1.45	0.28 ± 0.01	3
**QVLQQSTYQLLQELCCQHLW**	++	7.49	0.33	1
**LQELCCQHLWQIPEQSQCQA**	++	8.85	0.18	1
**QIPEQSQCQAIHNVVHAIIL**	+++	8.64	0.33	1
IHNVVHAIILHQQQKQQQQP	-			1
HQQQKQQQQPSSQVSFQQPL	-			1
**SSQVSFQQPLQQYPLGQGSF**	++	7.65	0.19	1
**QQYPLGQGSFRPSQQNPLAQ**	++	8.08	0.21	1
RPSQQNPLAQGSVQPQQLPQ	-			1
**GSVQPQQLPQFEEIRNLALQ**	+++	8.10 ± 0.18	0.34 ± 0.01	3
FEEIRNLALQTLPAMCNVYI	-			1
**TLPAMCNVYIPPYCTIVPFG**	+++	6.94 ± 0.16	0.42 ± 0.08	2
**PPYCTIVPFGIFGTNYR**	++	8.55 ± 0.11	0.29 ± 0.01	2

In a second series of experiments, the migration ability of four of the thirteen alpha-gliadin peptides was tested in the presence of cyclosporine H. We found that FPR1 antagonism completely abrogated neutrophil migration to these peptide motifs, confirming both the specificity of the gliadin-induced neutrophil migratory response and binding to FPR1 (Table 3). Application of a pool consisting a mixture of these four peptides enhanced the chemotactic response (with a speed of 9.96 μm/min and a chemotactic index of 0.54) but did not yet reach the strength of the chemotactic response induced by PT-gliadin (speed: 12.41 μm/min, CI: 0.73). Together, these findings show that synthetic gliadin peptides induce neutrophil migration in an FPR1-dependent fashion.

**Table 3:** Blocking of FPR1 inhibits neutrophil migration to gliadin peptides. Four alpha-gliadin synthetic peptides that displayed a chemotactic response were elected and tested in the presence of cyclosporine H, a specific inhibitor of FPR1. Blocking of FPR1 inhibited neutrophil chemotaxis to these peptides.

**Peptide**	**Speed (μm/minute)**		***P*-value**	**Number of experiments**
	Control	Cyclosporine H		
fMet-Leu-Phe	9.80 ± 0.46	0.35 ± 0.05	*P*<.001	3
QQHNIAHGRSQVLQQSTYQL	6.65 ± 0.33	0.48 ± 0.19	*P*<.001	3
TLPAMCNVYIPPYCTIVPFG	6.33 ± 1.10	0.34 ± 0.04	*P* = .016	2
MVRVPVPQLQPQNPSQQHPQ	7.50 ± 1.95	0.45 ± 0.11	*P* = .004	3
GSVQPQQLPQFEEIRNLALQ	7.90 ± 0.87	0.30 ± 0.06	*P* = .007	2
**Peptide**	**Chemotaxis Index (CI)**		***P*-value**	**Number of experiments**
	Control	Cyclosporine H		
fMet-Leu-Phe	0.76 ± 0.02	0.03 ± 0.01	*P*<.001	3
QQHNIAHGRSQVLQQSTYQL	0.27 ± 0.02	0.05 ± 0.02	*P* = .005	3
TLPAMCNVYIPPYCTIVPFG	0.33 ± 0.04	0.03 ± 0.01	*P* = .011	2
MVRVPVPQLQPQNPSQQHPQ	0.28 ± 0.03	0.03 ± 0.01	*P* = .020	3
GSVQPQQLPQFEEIRNLALQ	0.33 ± 0.07	0.02 ± 0.01	*P* = .043	2

**Fig 5:**
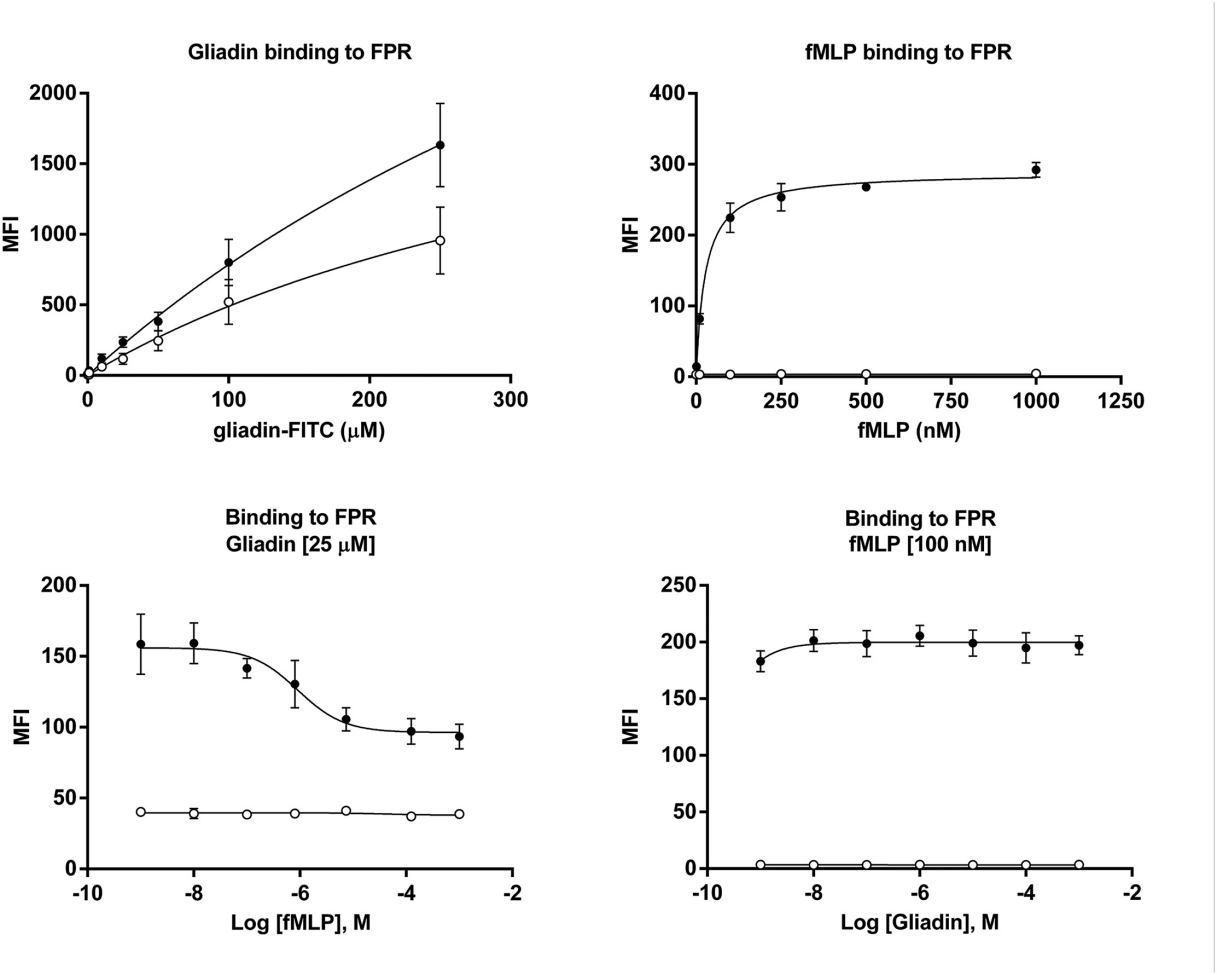
Binding of fMet-Leu-Phe and PT-gliadin to FPR1. Flow cytometry analysis of FITC-labeled gliadin peptide or fMet-Leu-Phe binding to FPR1-transfected or non-transfected HEK293T cells. (A) Kinetic binding of FITC-labeled gliadin synthetic peptide, TLPAMCNVYIPPYCTIVPFG, applied at increasing concentrations (ranging from 1 to 250 μM). Dissociation constant (K_d_) was 235.1 μM, and Bmax was 922.4. (B) Kinetic binding of FITC-labeled fMet-Leu-Phe, applied at increasing concentrations (ranging from 1 to 1000 nM). Dissociation constant (K_d_) was 27.6 nM, and Bmax was 261.6. (C) Competitive binding assay was performed with FITC-labeled gliadin peptide at 25.6 μM and unlabeled fMet-Leu-Phe at increasing concentrations (ranging from 10 nM to 2 mM). fMet-Leu-Phe caused a displacement of the gliadin peptide from the FPR1-transfected HEK293T cells with an IC50 of 2.04 μM. (D) Competitive binding assay was performed with FITC-labeled fMet-Leu-Phe at 100 nM and unlabeled gliadin peptide at increasing concentrations (ranging from 1 nM to 500 μM). The gliadin peptide was not capable of displacement of the fMet-Leu-Phe from the FPR1-transfected HEK293T cells. Binding to non-transfected cells and FPR1-transfected HEK293T cells is depicted with white and black circles, respectively. Each graph represents data from 3–5 independent experiments.

To demonstrate that gliadin binds to FPR1, we performed binding kinetic experiments on FPR1-transfected HEK293T cells with fMet-Leu-Phe and one of the gliadin peptides that induced neutrophil migration in the EZtaxi-scan assay, TLPAMCNVYIPPYCTIVPFG (Tables 2 and 3). The results showed a dissociation constant (K_d_) of 27.6 nM and a Bmax of 261.6 for fMet-Leu-Phe. Dissociation constant (K_d_) and Bmax for the gliadin peptide were 235.1 μM and 922.4, respectively (Fig 5A and 5B). We then performed a competitive binding assay where fMet-Leu-Phe, added in increasing concentrations, caused a displacement of the gliadin peptide (25 μM) from the FPR1-transfected HEK293T cells with an IC50 of 2.04 μM (C). However, in the competitive binding assay, where the gliadin peptide was added in increasing concentrations, the peptide could not compete away fMet-Leu-Phe (100 nM) (Fig 5D). Together, these results suggest that the gliadin peptide binds to FPR1 with low affinity and that gliadin’s binding to FPR1 is not entirely specific.

## Discussion

Gliadin affects the gut epithelial barrier function by causing a transient, spike-wise increased gut permeability in healthy individuals and a sustained compromised epithelial barrier function in individuals at risk for developing CD [15] as well as during the active phase of the disease [31, 32]. Our data confirm that upon exposure to the intestinal epithelium, gliadin causes a rapid redistribution of the major junctional protein, ZO-1, a characteristic feature of impaired intestinal barrier function. It is conceivable that the increased intestinal permeability allows access of gliadin to the *lamina propria*, but little is known about the subsequent early immune events.

We show here that gliadin exhibits chemoattractant properties and directly recruits neutrophils. This is a specific characteristic of gliadin, since zein was not capable of inducing neutrophil migration. Furthermore, a possible migratory effect of residual traces of enzymatic activity within the preparations was excluded by testing a digested water or zein control. In addition to rigorous and certified endotoxin testing of our preparations, we also included alpha-gliadin synthetic peptide motifs in our experimental design.

Our results demonstrate that neutrophil recruitment is a very early phenomenon that starts within 30 minutes after gliadin challenge. Gliadin appears to act as a neutrophil chemoattractant factor of similar potency to fMet-Leu-Phe and indeed appears to do so by binding to FPR1. The kinetic binding assays suggest that gliadin peptides bind to FPR1 albeit with low affinity. Given that the peptide could not compete higher concentrations of fMet-Leu-Phe, the binding may not be entirely specific. For this reason, we cannot exclude that gliadin peptides could regulate FPR1 indirectly. In addition to the PT-gliadin mixture, synthetic peptides spanning alpha-gliadin were individually tested for their capacity to induce neutrophil migration. Although the speed of neutrophil migration towards the peptides was comparable to that induced by the PT-gliadin mixture, the individual peptides induced a weaker directional migration, suggesting that the peptides generated a prevalent chemokinetic response.

Both chemotactic (where the cells move towards a gradient) and chemokinetic migration patterns (where the cells are activated without a gradient towards which to move) have been described in various reports and often as parallel observations but the biological significance of chemokinesis is largely unknown [33–35]. One possible explanation for the prevalent chemokinetic behavior of the peptides versus the chemotactic behavior of the gliadin mixture might be due to the use of non-optimal concentrations of the discrete peptides. Another possible explanation may be provided by the described two-step migratory event [35, 36], in which the chemokinetic signal is considered to be the activation of “locomotive potency” that is followed by the “direction-oriented” chemotactic signals. A third explanation is the loss of synergistic action of the peptides within the PT-gliadin mixture when single peptides are applied. This latter hypothesis is reinforced by the finding that four pooled peptides exerted a stronger chemotactic response than the single peptides, even if the response did not equal the strength of the chemotactic effect that was induced by the PT-gliadin mixture.

Although both mouse and human neutrophils migrated to fMet-Leu-Phe and PT-gliadin, mouse neutrophils were much less responsive. This is in line with various reports [37, 38] demonstrating that fMet-Leu-Phe is a much less potent agonist for mouse FPR1, which of all eight members of the mouse FPR family is closest to its human counterpart, than it is for human FPR1. Since we observed a rapid extravasation of neutrophils in response to luminal gliadin exposure and an optimal tissue influx in the *lamina propria* twenty-four hours after gavage, we may anticipate an even more robust neutrophil recruitment in humans *in vivo*.

Combined with earlier studies, our results show that gliadin may induce in any individual, regardless of the condition, an at least temporary disassembly of intestinal TJ [15], neutrophil migration, and cytokine production [18, 39]. Even if neutrophils have traditionally not been associated with CD pathogenesis, histological examination of biopsy specimens from a significant subset of CD patients shows an increased presence of neutrophils in the intestinal tissue. Some reports have described neutrophils influx by measurement of their marker, myeloperoxidase, within 3–5 hours upon rectal gluten challenge [24, 25]. A recent report mentioned an increased number of neutrophils in the celiac mucosa after a three-day gluten challenge [40], and Diosdado et al. [41] showed ongoing neutrophil activation and recruitment to the gut even after implementation of a gluten-free diet and normalization of serology, suggesting that a primary barrier defect is at the base of the ongoing neutrophil presence.

In conclusion, the present study emphasizes the emerging concept that PT-gliadin is interpreted and handled by the host mucosa as a danger signal similar to that provided by bacterial bio-products, e.g. fMet-Leu-Phe, likewise exerting direct and robust chemoattractant effects on neutrophils. These observations provide new insight into our understanding of how gliadin triggers inflammatory responses. To what extent neutrophil function adds to, or protects against, gluten intolerance is currently under vigorous investigation.

## Supporting Information

S1 Fig Schematic figure of alpha-gliadin. Thirteen out of 25 peptide sequences were capable of inducing neutrophil migration. This figure depicts the localization of these peptides within the alpha-gliadin protein. Indicated in yellow are the peptide motifs that induced a moderate (++) response and in red those that induced stronger chemotactic response (+++). See also Table 2.
(TIF)

S1 Movie PT-gliadin attracts neutrophils. PT-gliadin was injected in a 5 cm long loop of Lys-GFP transgenic mice duodenum. Differently from the PBS injected loop (S1B movie), neutrophils appear to roll more slowly along the vessels. Several neutrophils stop and seem to invade the intestinal tissue. Finally some areas of the imaged tissue reveal bright spots indicating neutrophil invasion.
(MP4)

S2 Movie PBS does not attract neutrophils. The PBS injected loop did not show any neutrophil recruitment.
(MP4)

S3 Movie Murine neutrophil chemotaxis to PT-gliadin using EZ-TAXIScan chemotactic assay. The EZ-TAXIScan chemotactic assay [22] was used for direct visualization of murine neutrophil chemotaxis to PT-gliadin. N-formyl-Methionine-Leucine-Phenylalanine (fMet-Leu-Phe, middle panel) was used as a positive control and PT-digested water was used as a negative control. PT-gliadin (lower panel) was at least as effective as the primary chemoattractant, fMet-Leu-Phe at inducing chemotaxis of bone marrow-derived mouse neutrophils. Neutrophils did not migrate towards the negative control (upper panel). Frames were taken every 15 seconds.
(MOV)

S4 Movie Human neutrophil chemotaxis to PT-gliadin using EZ-TAXIScan chemotactic assay. The EZ-TAXIScan chemotactic assay was used for direct visualization of human neutrophil migration to either PT-gliadin or fMet-Leu-Phe. PT-digested water and PT-zein were used as a negative control. As observed in mice, PT-gliadin and fMet-Leu-Phe induced similar chemotactic responses. Neutrophils did not migrate towards the PT-digested water and zein control. Frames were taken every 15 seconds.
(AVI)

S5 Movie CXCR3^-/-^ neutrophil chemotaxis using EZ-TAXIScan chemotactic assay. The EZ-TAXIScan chemotactic assay was used for direct visualization of chemotaxis of CXCR3^-/-^ neutrophils to either PT-gliadin (lower panel) or fMet-Leu-Phe (middle panel). PBS (upper panel) was used as a negative control. PT-gliadin and fMet-Leu-Phe induced similar chemotactic responses as C57BL/6 wild-type neutrophils (see S2 movie), indicating that neutrophil chemotaxis is independent of PT-gliadin binding to CXCR3. Frames were taken every 15 seconds.
(MOV)

S1 Table Alpha-gliadin synthetic peptide library. The alpha-gliadin synthetic peptide library contains 25 20-mer, 10-mer overlapping, peptides. The peptide sequences in red were capable of inducing neutrophil migration.
(PDF)
