# Supplementary figures and images for: Gliadin Induces Neutrophil Migration via Engagement of the Formyl Peptide Receptor, FPR1
Source: PLoS One. 2015 Sep 17;10(9):e0138338. doi: 10.1371/journal.pone.0138338 (PMC4574934; doi:10.1371/journal.pone.0138338)

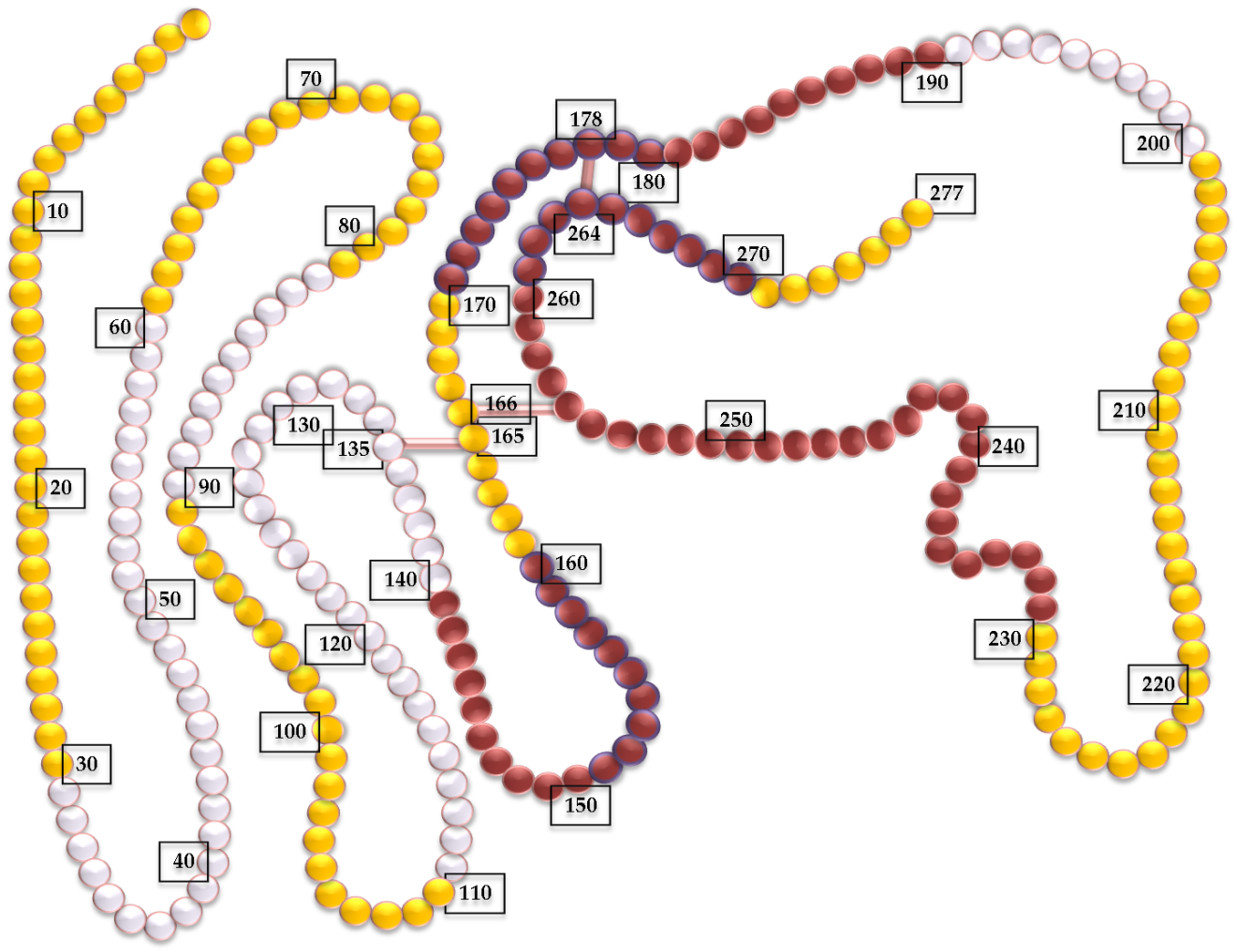

Supplement: S1 Fig — Thirteen out of 25 peptide sequences were capable of inducing neutrophil migration. This figure depicts the localization of these peptides within the alpha-gliadin protein. Indicated in yellow are the peptide motifs that induced a moderate (++) response and in red those that induced stronger chemotactic response (+++). See also Table 2. (TIF) [file pone.0138338.s001.tif]
