# Supplementary material for: Gliadin Induces Neutrophil Migration via Engagement of the Formyl Peptide Receptor, FPR1
Source: PLoS One. 2015 Sep 17;10(9):e0138338. doi: 10.1371/journal.pone.0138338 (PMC4574934; doi:10.1371/journal.pone.0138338)
Supplement: S1 Table — The alpha-gliadin synthetic peptide library contains 25 20-mer, 10-mer overlapping, peptides. The peptide sequences in red were capable of inducing neutrophil migration. (PDF) [file pone.0138338.s007.pdf]

MVRVPVPQLQPQNPSQQHPQ
PQNPSQQHPQEQVPLVQQQQ
EQVPLVQQQQFLGQQQSFPP
FLGQQQSFPPQQPYPQPQPF

QPYPQPQPFPSQQPYLQLQ
PSQQPYLQLQPFPQPQLPYL
PFPQPQLPYLQPQPFRPQQP
QPQPFRPQQPYPQPQPQYSQ

YPQPQPQYSQPQQPISQQQQ
PQQPISQQQQQQQQQQQQQQ
QQQQQQQQQQQQQQQQQQQQ
QQQQQQQQQQQILQQILQQ

QQILQQILQQQLIPCMDVVL
QLIPCMDVVLQQHNIAHGRS
QQHNIAHGRSQVLQQSTYQL
QVLQQSTYQLLQELCCQHLW

LQELCCQHLWQIPEQSQCQA
QIPEQSQCQAIHNVVHAIIL
IHNVVHAIILHQQQKQQQQP
HQQQKQQQQPSSQVSFQQPL

SSQVSFQQPLQQYPLGQGSF
QQYPLGQGSFRPSQQNPLAQ
RPSQQNPLAQGSVQPQQLPQ
GSVQPQQLPQFEEIRNLALQ

FEEIRNLALQTLPAMCNVYI
TLPAMCNVYIPPYCTIVPFG
PPYCTIVPFGIFGTNYR
